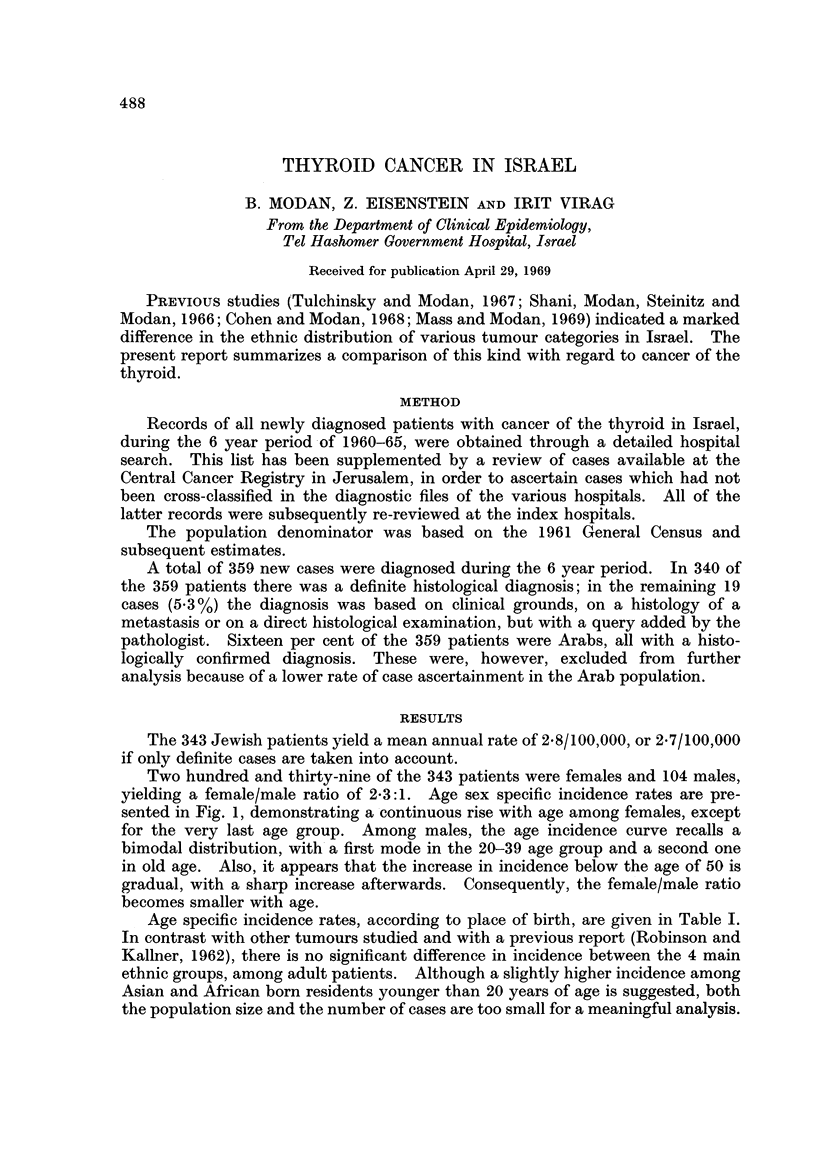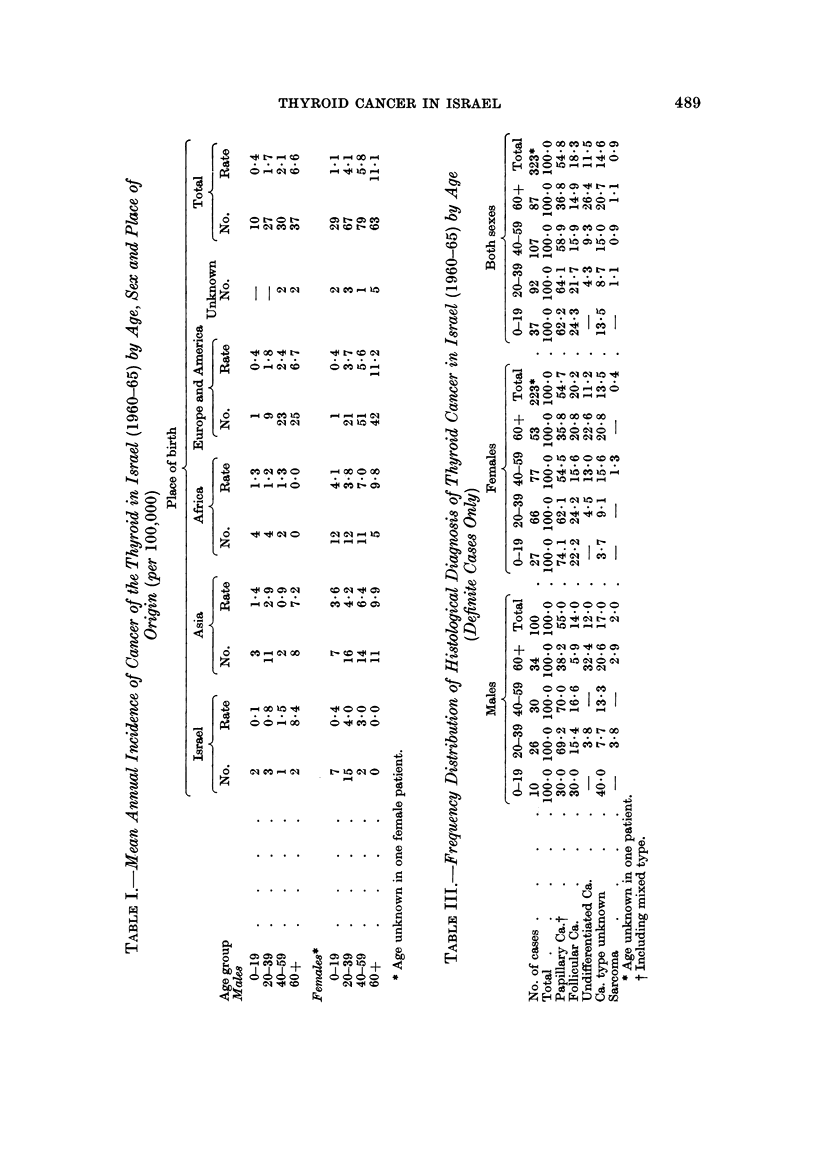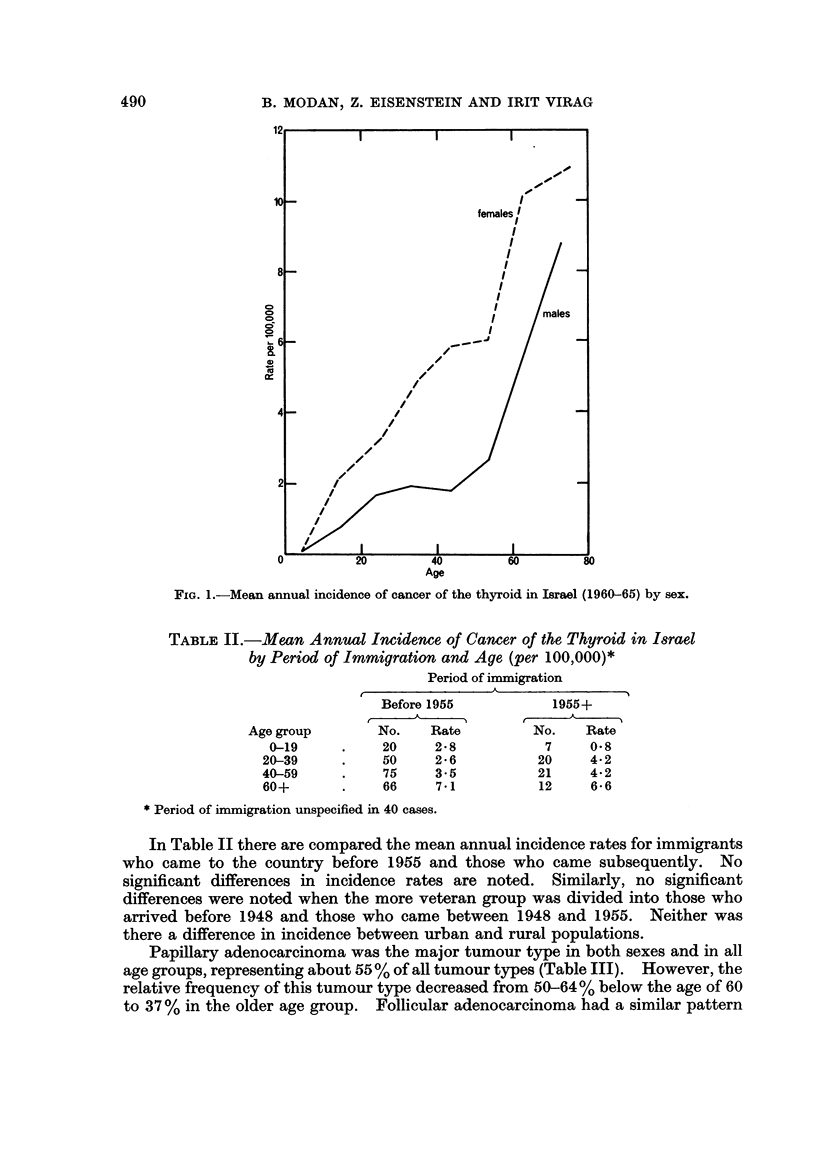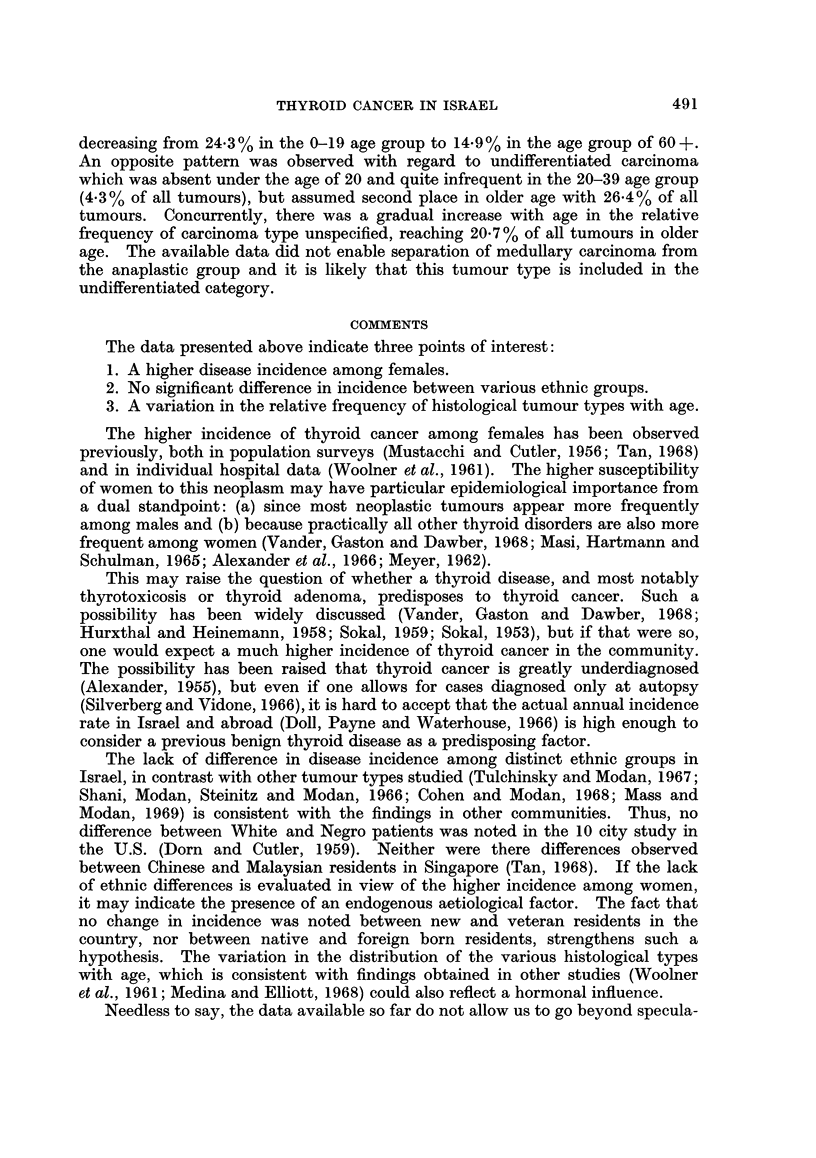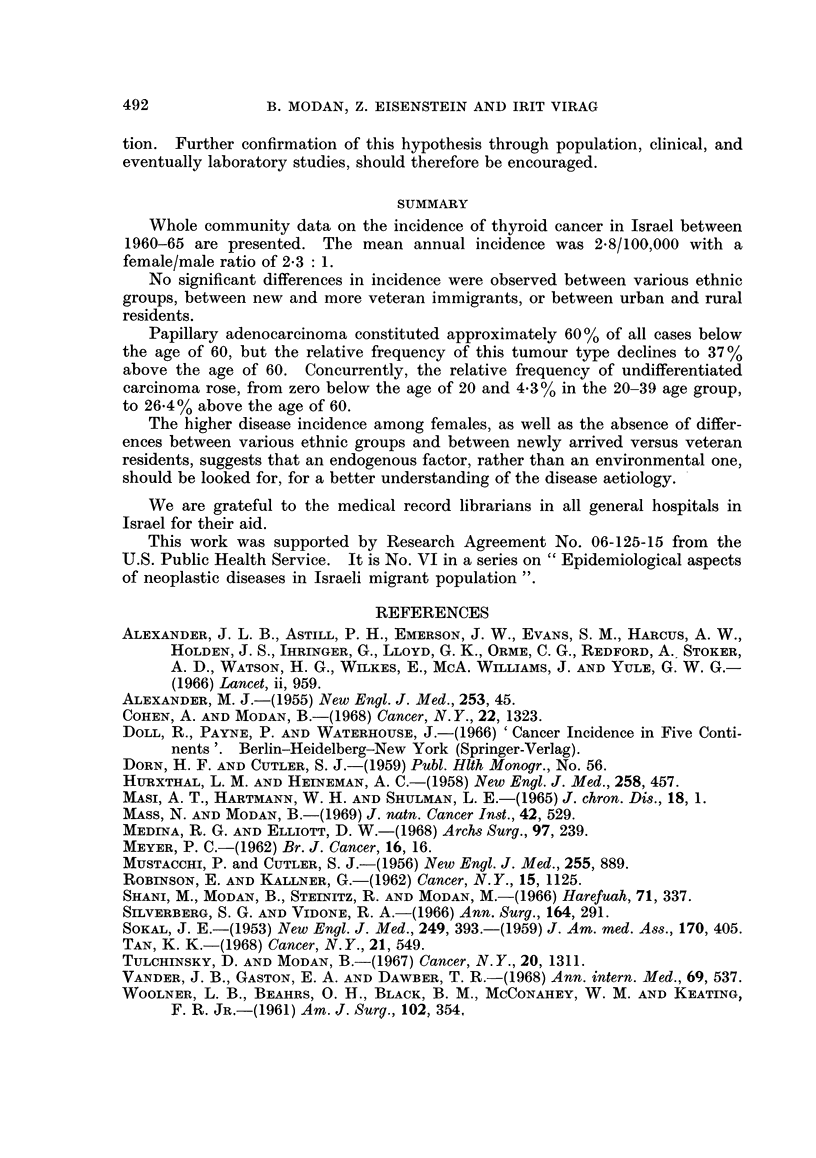# Thyroid cancer in Israel.

**DOI:** 10.1038/bjc.1969.61

**Published:** 1969-09

**Authors:** B. Modan, M. Z. Eisenstein, I. Virag


### THYROID CANCER IN ISRAEL

B. MODAN, Z. EISENSTEIN AND IRIT VIRAG
*From the Department of Clinical Epidemiology, Tel Hashomer Government Hospital, Israel*

Received for publication April 29, 1969

PREVIOUS studies (Tulchinsky and Modan, 1967; Shani, Modan, Steinitz and Modan, 1966; Cohen and Modan, 1968; Mass and Modan, 1969) indicated a marked difference in the ethnic distribution of various tumour categories in Israel. The present report summarizes a comparison of this kind with regard to cancer of the thyroid.

### METHOD

Records of all newly diagnosed patients with cancer of the thyroid in Israel, during the 6 year period of 1960–65, were obtained through a detailed hospital search. This list has been supplemented by a review of cases available at the Central Cancer Registry in Jerusalem, in order to ascertain cases which had not been cross-classified in the diagnostic files of the various hospitals. All of the latter records were subsequently re-reviewed at the index hospitals.

The population denominator was based on the 1961 General Census and subsequent estimates.

A total of 359 new cases were diagnosed during the 6 year period. In 340 of the 359 patients there was a definite histological diagnosis; in the remaining 19 cases (5·3%) the diagnosis was based on clinical grounds, on a histology of a metastasis or on a direct histological examination, but with a query added by the pathologist. Sixteen per cent of the 359 patients were Arabs, all with a histologically confirmed diagnosis. These were, however, excluded from further analysis because of a lower rate of case ascertainment in the Arab population.

### RESULTS

The 343 Jewish patients yield a mean annual rate of 2·8/100,000, or 2·7/100,000 if only definite cases are taken into account.

Two hundred and thirty-nine of the 343 patients were females and 104 males, yielding a female/male ratio of 2·3:1. Age sex specific incidence rates are presented in Fig. 1, demonstrating a continuous rise with age among females, except for the very last age group. Among males, the age incidence curve recalls a bimodal distribution, with a first mode in the 20–39 age group and a second one in old age. Also, it appears that the increase in incidence below the age of 50 is gradual, with a sharp increase afterwards. Consequently, the female/male ratio becomes smaller with age.

Age specific incidence rates, according to place of birth, are given in Table I. In contrast with other tumours studied and with a previous report (Robinson and Kallner, 1962), there is no significant difference in incidence between the 4 main ethnic groups, among adult patients. Although a slightly higher incidence among Asian and African born residents younger than 20 years of age is suggested, both the population size and the number of cases are too small for a meaningful analysis.

FIG. 1.—Mean annual incidence of cancer of the thyroid in Israel (1960–65) by sex.

TABLE II.—*Mean Annual Incidence of Cancer of the Thyroid in Israel by Period of Immigration and Age* (*per* 100,000)*

| | Period of immigration | | | |
|---|---|---|---|---|
| | Before 1955 | | 1955+ | |
| Age group | No. | Rate | No. | Rate |
| 0–19 . | 20 | 2·8 | 7 | 0·8 |
| 20–39 . | 50 | 2·6 | 20 | 4·2 |
| 40–59 . | 75 | 3·5 | 21 | 4·2 |
| 60+ . | 66 | 7·1 | 12 | 6·6 |

* Period of immigration unspecified in 40 cases.

In Table II there are compared the mean annual incidence rates for immigrants who came to the country before 1955 and those who came subsequently. No significant differences in incidence rates are noted. Similarly, no significant differences were noted when the more veteran group was divided into those who arrived before 1948 and those who came between 1948 and 1955. Neither was there a difference in incidence between urban and rural populations.

Papillary adenocarcinoma was the major tumour type in both sexes and in all age groups, representing about 55% of all tumour types (Table III). However, the relative frequency of this tumour type decreased from 50–64% below the age of 60 to 37% in the older age group. Follicular adenocarcinoma had a similar pattern decreasing from 24·3% in the 0–19 age group to 14·9% in the age group of 60+. An opposite pattern was observed with regard to undifferentiated carcinoma which was absent under the age of 20 and quite infrequent in the 20–39 age group (4·3% of all tumours), but assumed second place in older age with 26·4% of all tumours. Concurrently, there was a gradual increase with age in the relative frequency of carcinoma type unspecified, reaching 20·7% of all tumours in older age. The available data did not enable separation of medullary carcinoma from the anaplastic group and it is likely that this tumour type is included in the undifferentiated category.

### COMMENTS

The data presented above indicate three points of interest:

1. A higher disease incidence among females.
2. No significant difference in incidence between various ethnic groups.
3. A variation in the relative frequency of histological tumour types with age.

The higher incidence of thyroid cancer among females has been observed previously, both in population surveys (Mustacchi and Cutler, 1956; Tan, 1968) and in individual hospital data (Woolner *et al.*, 1961). The higher susceptibility of women to this neoplasm may have particular epidemiological importance from a dual standpoint: (a) since most neoplastic tumours appear more frequently among males and (b) because practically all other thyroid disorders are also more frequent among women (Vander, Gaston and Dawber, 1968; Masi, Hartmann and Schulman, 1965; Alexander *et al.*, 1966; Meyer, 1962).

This may raise the question of whether a thyroid disease, and most notably thyrotoxicosis or thyroid adenoma, predisposes to thyroid cancer. Such a possibility has been widely discussed (Vander, Gaston and Dawber, 1968; Hurxthal and Heinemann, 1958; Sokal, 1959; Sokal, 1953), but if that were so, one would expect a much higher incidence of thyroid cancer in the community. The possibility has been raised that thyroid cancer is greatly underdiagnosed (Alexander, 1955), but even if one allows for cases diagnosed only at autopsy (Silverberg and Vidone, 1966), it is hard to accept that the actual annual incidence rate in Israel and abroad (Doll, Payne and Waterhouse, 1966) is high enough to consider a previous benign thyroid disease as a predisposing factor.

The lack of difference in disease incidence among distinct ethnic groups in Israel, in contrast with other tumour types studied (Tulchinsky and Modan, 1967; Shani, Modan, Steinitz and Modan, 1966; Cohen and Modan, 1968; Mass and Modan, 1969) is consistent with the findings in other communities. Thus, no difference between White and Negro patients was noted in the 10 city study in the U.S. (Dorn and Cutler, 1959). Neither were there differences observed between Chinese and Malaysian residents in Singapore (Tan, 1968). If the lack of ethnic differences is evaluated in view of the higher incidence among women, it may indicate the presence of an endogenous aetiological factor. The fact that no change in incidence was noted between new and veteran residents in the country, nor between native and foreign born residents, strengthens such a hypothesis. The variation in the distribution of the various histological types with age, which is consistent with findings obtained in other studies (Woolner *et al.*, 1961; Medina and Elliott, 1968) could also reflect a hormonal influence.

Needless to say, the data available so far do not allow us to go beyond speculation. Further confirmation of this hypothesis through population, clinical, and eventually laboratory studies, should therefore be encouraged.

### SUMMARY

Whole community data on the incidence of thyroid cancer in Israel between 1960–65 are presented. The mean annual incidence was 2·8/100,000 with a female/male ratio of 2·3 : 1.

No significant differences in incidence were observed between various ethnic groups, between new and more veteran immigrants, or between urban and rural residents.

Papillary adenocarcinoma constituted approximately 60% of all cases below the age of 60, but the relative frequency of this tumour type declines to 37% above the age of 60. Concurrently, the relative frequency of undifferentiated carcinoma rose, from zero below the age of 20 and 4·3% in the 20–39 age group, to 26·4% above the age of 60.

The higher disease incidence among females, as well as the absence of differences between various ethnic groups and between newly arrived versus veteran residents, suggests that an endogenous factor, rather than an environmental one, should be looked for, for a better understanding of the disease aetiology.

We are grateful to the medical record librarians in all general hospitals in Israel for their aid.

This work was supported by Research Agreement No. 06-125-15 from the U.S. Public Health Service. It is No. VI in a series on " Epidemiological aspects of neoplastic diseases in Israeli migrant population ".

### REFERENCES

Alexander, J. L. B., Astill, P. H., Emerson, J. W., Evans, S. M., Harcus, A. W., Holden, J. S., Ihringer, G., Lloyd, G. K., Orme, C. G., Redford, A., Stoker, A. D., Watson, H. G., Wilkes, E., McA. Williams, J. and Yule, G. W. G.—(1966) *Lancet*, ii, 959.

Alexander, M. J.—(1955) *New Engl. J. Med.*, **253**, 45.

Cohen, A. and Modan, B.—(1968) *Cancer, N.Y.*, **22**, 1323.

Doll, R., Payne, P. and Waterhouse, J.—(1966) ' Cancer Incidence in Five Continents '. Berlin–Heidelberg–New York (Springer-Verlag).

Dorn, H. F. and Cutler, S. J.—(1959) *Publ. Hlth Monogr.*, No. 56.

Hurxthal, L. M. and Heineman, A. C.—(1958) *New Engl. J. Med.*, **258**, 457.

Masi, A. T., Hartmann, W. H. and Shulman, L. E.—(1965) *J. chron. Dis.*, **18**, 1.

Mass, N. and Modan, B.—(1969) *J. natn. Cancer Inst.*, **42**, 529.

Medina, R. G. and Elliott, D. W.—(1968) *Archs Surg.*, **97**, 239.

Meyer, P. C.—(1962) *Br. J. Cancer*, **16**, 16.

Mustacchi, P. and Cutler, S. J.—(1956) *New Engl. J. Med.*, **255**, 889.

Robinson, E. and Kallner, G.—(1962) *Cancer, N.Y.*, **15**, 1125.

Shani, M., Modan, B., Steinitz, R. and Modan, M.—(1966) *Harefuah*, **71**, 337.

Silverberg, S. G. and Vidone, R. A.—(1966) *Ann. Surg.*, **164**, 291.

Sokal, J. E.—(1953) *New Engl. J. Med.*, **249**, 393.—(1959) *J. Am. med. Ass.*, **170**, 405.

Tan, K. K.—(1968) *Cancer, N.Y.*, **21**, 549.

Tulchinsky, D. and Modan, B.—(1967) *Cancer, N.Y.*, **20**, 1311.

Vander, J. B., Gaston, E. A. and Dawber, T. R.—(1968) *Ann. intern. Med.*, **69**, 537.

Woolner, L. B., Beahrs, O. H., Black, B. M., McConahey, W. M. and Keating, F. R. Jr.—(1961) *Am. J. Surg.*, **102**, 354.